# Tumor-derived extracellular vesicles regulate tumor-infiltrating regulatory T cells via the inhibitory immunoreceptor CD300a

**DOI:** 10.7554/eLife.61999

**Published:** 2021-11-09

**Authors:** Yuta Nakazawa, Nanako Nishiyama, Hitoshi Koizumi, Kazumasa Kanemaru, Chigusa Nakahashi-Oda, Akira Shibuya

**Affiliations:** 1 Department of Immunology, Faculty of Medicine, University of Tsukuba Tsukuba Japan; 2 Doctoral Program of Biomedical Sciences, Graduate School of Comprehensive Human Sciences, University of Tsukuba Tsukuba Japan; 3 R&D Center for Innovative Drug Discovery, University of Tsukuba Tsukuba Japan; 4 Life Science Center for Survival Dynamics, Tsukuba Advanced Research Alliance (TARA), University of Tsukuba Tsukuba Japan; Osaka University Japan; Institute of Industrial Science, The University of Tokyo Japan

**Keywords:** tumor-infiltrating regulatory T cells, tumor-derived extracellular vesicles, immunoreceptor, dendritic cells, IFN-b, CD300a, Mouse

## Abstract

Although tumor-infiltrating regulatory T (Treg) cells play a pivotal role in tumor immunity, how Treg cell activation are regulated in tumor microenvironments remains unclear. Here, we found that mice deficient in the inhibitory immunoreceptor CD300a on their dendritic cells (DCs) have increased numbers of Treg cells in tumors and greater tumor growth compared with wild-type mice after transplantation of B16 melanoma. Pharmacological impairment of extracellular vesicle (EV) release decreased Treg cell numbers in CD300a-deficient mice. Coculture of DCs with tumor-derived EV (TEV) induced the internalization of CD300a and the incorporation of EVs into endosomes, in which CD300a inhibited TEV-mediated TLR3–TRIF signaling for activation of the IFN-β-Treg cells axis. We also show that higher expression of CD300A was associated with decreased tumor-infiltrating Treg cells and longer survival time in patients with melanoma. Our findings reveal the role of TEV and CD300a on DCs in Treg cell activation in the tumor microenvironment.

## Introduction

CD4^+^ regulatory T (Treg) cells specifically expressing Foxp3 play an essential role for maintaining peripheral tolerance, preventing autoimmunity, and limiting chronic inflammatory diseases. Deficiency in Treg cells due to genetic inactivation of *Foxp3* or impaired induction of Treg cells after birth results in lethal autoinflammatory syndromes (Kim et al., 2007; Ramsdell and Ziegler, 2014). Treg cells are found at various tissues, including tumors, at various frequencies. Because tumor-infiltrating Treg cells suppress the activation of tumor antigen-specific CD8^+^ T cells, a greater proportion of Treg cells to CD8^+^ T cells among tumor-infiltrating lymphocytes is associated with poor prognosis in several cancers (Nishikawa and Sakaguchi, 2010). Indeed, Treg cell depletion dramatically reduces tumor burden (Klages et al., 2010). Current clinical trials are evaluating strategies targeting receptors (CD25, CTLA-4, CCR4, OX40, and GITR) preferentially expressed on intratumoral Treg cells (Nishikawa and Sakaguchi, 2010; Shitara and Nishikawa, 2018). The migration of Treg cells and their activation and proliferation are regulated by chemoattractants (Adeegbe and Nishikawa, 2013; Ondondo et al., 2013) and cytokines such as TGF-β and IL-10 (Hsu et al., 2015; Wan and Flavell, 2007). However, how Treg cell activation and proliferation are regulated in the tumor microenvironments remains unclear.

Extracellular vesicles (EVs) are the particles released from the cell that are delimited by a lipid bilayer containing functional biomolecules (proteins, lipids, mRNAs, microRNAs, and DNA fragments) that can be transferred to other cells (van Niel et al., 2018; Witwer and Théry, 2019). More than 4000 trillion EVs are presumed to be in the blood of cancer patients (Melo et al., 2015) and EVs released from tumor cells (tumor-derived EVs [TEVs]) are emerging as critical messengers in tumor progression and metastasis (Couto et al., 2018; Grange et al., 2011; Melo et al., 2015; Skog et al., 2008). In tumor immunity, pleiotropic and deleterious role of TEV has been reported that, Fas ligand and PD-L1, the immunomodulatory molecules, on the surface of TEV induce apoptosis or suppression of activated T cells (Andreola et al., 2002; Chen et al., 2018) and TGF-β1 in TEV induces Treg cells (Clayton et al., 2007). TEV also upregulates PD-L1 expression on myeloid cells (Fleming et al., 2019). Furthermore, myeloid cells that capture microRNA within TEVs are altered to myeloid-derived suppressor cells and/or M2 macrophages and promote the malignant behavior of cancers (Huber et al., 2018; Tian et al., 2019; Wang et al., 2018; Ying et al., 2016). These intensive investigations have shown that TEVs play a key role in the suppression of antitumor immune responses (Zebrowska et al., 2020). However, how TEV regulates myeloid cell activation in tumor microenvironment is still incompletely understood.

The mouse CD300 family molecules, which are encoded by nine genes on chromosome 11, are expressed on myeloid cells including macrophages, dendritic cells (DCs), mast cells, and granulocytes and either activate or inhibit innate immune responses (Borrego, 2013; Voss et al., 2015). On the other hand, the human CD300 family consists of seven molecules encoded by genes located on chromosome 17 in a region syntenic to mouse chromosome 11 (Clark et al., 2001). CD300a, one of the CD300 molecules in mouse, contains two immunoreceptor tyrosine-based inhibitory motifs in its cytoplasmic portion. It mediates an inhibitory signal via SHP-1 and SHP-2 by binding to phosphatidylserine, which is exposed on the outer leaflet of the plasma membrane on apoptotic cells and activated mast cells under degranulation (Nakahashi-Oda et al., 2012a; Wang et al., 2019; Yotsumoto et al., 2003). Upon binding to phosphatidylserine, CD300a inhibits TLR4-mediated signaling in mast cells and DCs, which results in the suppression of cytokine and chemokine production and modulation of inflammatory immune responses (Nakahashi-Oda et al., 2016; Nakahashi-Oda et al., 2012b).

Here, we investigated the role of CD300a in tumor development and demonstrate that CD300a inhibits TEV-mediated interferon-β (IFN-β) production by DCs and suppresses the activation of tumor-infiltrating Treg cells and tumor development.

## Results

### CD300a on DCs enhances antitumor immunity

**Figure 1.**
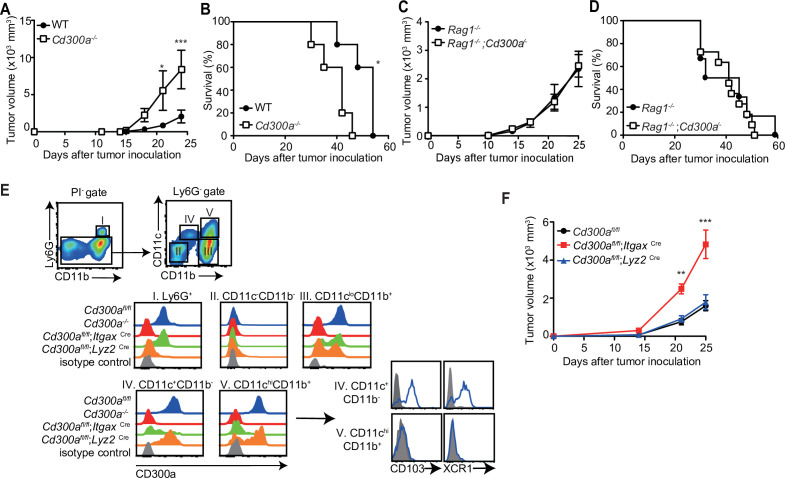
CD300a suppresses tumor growth. (**A–D**) Tumor growth or survival curves of wild-type (WT, *n* = 5 in **A** and **B**), *Cd300a^−/−^* (*n* = 5 in **A** and **B**), *Rag1^−/−^* (*n* = 11 in **C** and *n* = 6 in **D**), and *Rag1^−/−^;Cd300a^−/−^* (*n* = 15 in **C** and *n* = 11 in **D**) that were inoculated with 1 × 10^5^ B16 melanoma cells on day 0. (**E**) CD300a expression on neutrophils (Ly6G^+^), macrophages (Ly6G^−^CD11c^lo^CD11b^+^), conventional type-1 DC (cDC1; Ly6G^−^CD11c^+^CD11b^−^CD103^+^XCR1^+^), and cDC1 (Ly6G^−^CD11c^+hi^CD11b^+^CD103^−^XCR^−^) isolated from B16 melanoma tissues of *Cd300a*^fl/fl^, *Cd300a^−/−^*, *Cd300a*^fl/fl^;*Itgax*^Cre^, and *Cd300a*^fl/fl^;*Lys2*^Cre^ mice prepared 14 days after inoculation. Data are representative of three mice. (**F**) Tumor growth of *Cd300a*^fl/fl^ (*n* = 7), *Cd300a*^fl/fl^;*Itgax*^Cre^ (*n* = 13), and *Cd300a*^fl/fl^;*Lys2*^Cre^ mice (*n* = 15) that were inoculated with 1 × 10^5^ B16 melanoma cells on day 0. Data are given as means ± standard error of the means (SEMs). **p** < 0.01 and ***p < 0.001. p values were obtained by using a two-way analysis of variance (ANOVA) followed by Bonferroni’s post-test (**A, C, and F**) and the log-rank test (**B and D**). Data were pooled from two (**A– C and E**) or three (**D and F**) independent experiments. Figure 1—source data 1.Source data for Figure 1A-D and F.

To address whether CD300a is involved in tumor immunity, wild-type and CD300a-deficient (*Cd300a^−/−^*) mice were transplanted intradermally with B16 melanoma cells. The *Cd300a^−/−^* mice showed larger tumor volume and shorter survival than did wild-type mice (Figure 1A and B), indicating that CD300a suppresses the development of melanoma. In contrast, Rag-deficient (*Rag1^−/−^*) and *Rag1^−/−^;Cd300a^−/−^* mice showed comparable levels of tumor development and survival after injection of B16 melanoma cells (Figure 1C and D). These results indicate that the suppressive effect of CD300a on melanoma development is dependent on the adaptive immune response. However, we also observed that CD300a was not expressed on tumor-infiltrating lymphocytes but was broadly expressed on myeloid cells, including populations of Ly6G^+^ neutrophils, CD11c^+~high^ DCs, and CD11c^low^CD11b^+^ macrophages (Figure 1E). These results suggest that CD300a expressed on myeloid cells suppresses melanoma development via adaptive immune responses. To identify the CD300a-expressing myeloid cell population that is involved in melanoma suppression, we used *Cd300a*^fl/fl^;*Itgax*^Cre^ and *Cd300a*^fl/fl^;*Lyz2*^Cre^ mice. *Cd300a*^fl/fl^;*Itgax*^Cre^ mice expressed CD300a on Ly6G^+^ cells and CD11c^−^ cells, but not on CD11c^+~high^ cells (Figure 1E). In contrast, *Cd300a*^fl/fl^;*Lyz2*^Cre^ mice express CD300a on CD11c^+~high^ cells and the subpopulation of CD11c^low^ cells, but not on Ly6G^+^ cells (Figure 1E). Although tumor growth was comparable between *Cd300a*^fl/fl^;*Lyz2*^Cre^ and *Cd300a*^fl/fl^ mice, *Cd300a*^fl/fl^;*Itgax*^Cre^ mice showed greater tumor volume than did *Cd300a*^fl/fl^ mice (Figure 1F). These data implicated CD300a on DCs, rather than on neutrophils or macrophages, in inducing the adaptive immune response to inhibit tumor development.

### CD300a regulates tumor-infiltrating Treg cells

**Figure 2.**
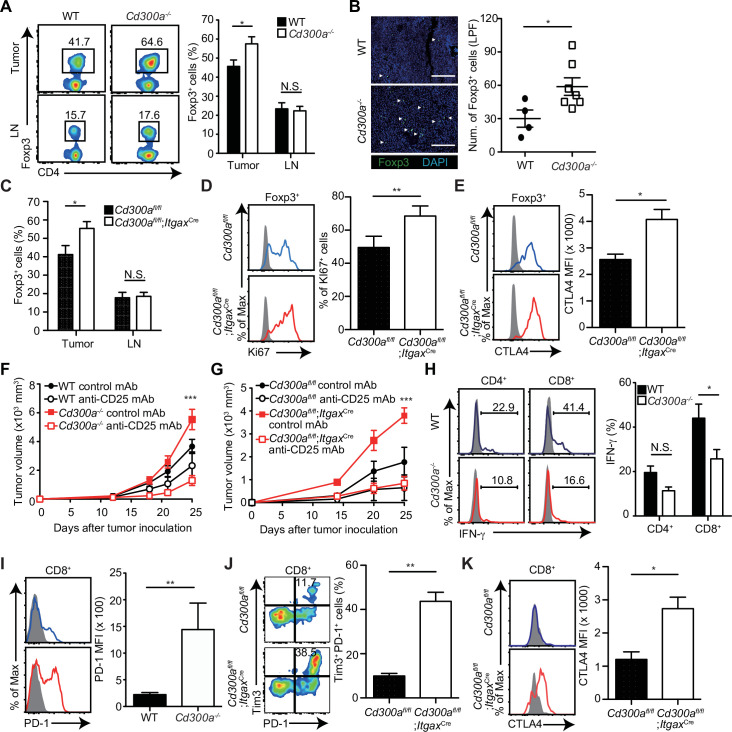
Tumor-infiltrating Treg cells are regulated by CD300a. Tumor tissues were harvested 3 weeks after B16 melanoma inoculation. (**A**) Representative flow cytometry plots of Treg cells in the tumor and draining lymph node (LN) (left). Numbers adjacent to outlined areas indicate the percentage of Foxp3^+^ (Treg) CD4^+^ cells. The frequencies of Foxp3^+^ cells among CD4^+^ T cells in both wild-type (WT, *n* = 7) and *Cd300a^−/−^* mice (*n* = 8) are shown (right). (**B**) Fluorescence microscopy of tumor sections from Foxp3-eGFP WT (*n* = 4) and *Cd300a^−/−^* (*n* = 7) mice, stained with an anti-GFP monoclonal antibody (green) and the DNA-binding dye 4′,6-diamidino-2-phenylindole (DAPI; left). The number of Foxp3^+^ cells was quantified from for low-power fields (LPF) (right). White arrow shows Foxp3-positive cells. Scale bar, 200 μm. (**C**) Flow cytometric analysis of the frequencies of Foxp3^+^ cells among CD4^+^ T cells in the tumor (*n* = 11 in each group) and draining lymph node (*n* = 8 in each group) in *Cd300a*^fl/fl^ and *Cd300a*^fl/fl^;*Itgax*^Cre^ mice. Flow cytometric analysis of Ki67 (**D**) and CTLA-4 (**E**) expressions of Treg cells in the tumor in *Cd300a*^fl/fl^ (*n* = 9 in D, *n* = 3 in E) and *Cd300a*^fl/fl^;*Itgax*^Cre^ mice (*n* = 10 in D, *n* = 4 in E). Representative histogram (left), frequency (right), (**D**), and mean fluorescent intensity (MFI, right, **E**). (**F and G**) Tumor growth curve of WT (control mAb, *n* = 7; anti-CD25 mAb, *n* = 5), *Cd300a^−/−^* (control mAb, *n* = 8; anti-CD25 mAb, *n* = 6), *Cd300a^fl/fl^* (control mAb, *n* = 4; anti-CD25 mAb, *n* = 3), and *Cd300a*^fl/fl^;*Itgax*^Cre^ (control mAb, *n* = 3; anti-CD25 mAb, *n* = 5) mice that were treated with an anti-CD25 mAb or a control antibody three times (days −6, −3, and 0) and then inoculated with B16 melanoma cells. (**H**) Representative histogram of IFN-γ production from tumor-infiltrating T cells after phorbol 12-myristate 13-acetate (PMA) and ionomycin stimulation (left). The proportion of IFN-γ^+^ cells is shown (right) (*n* = 6 in each group). (**I–K**) Flow cytometric analysis of the expressions of programmed cell death-1(PD-1) (**I**), PD-1 and Tim3 (**J**), and CTLA-4 (**K**) in CD8^+^ T cells in the tumor of *Cd300a*^fl/fl^ (*n* = 4 in I, *n* = 3 in J and K) and *Cd300a*^fl/fl^;*Itgax*^Cre^ (*n* = 6 in I, *n* = 5 in J, *n* = 4 in K) mice. Representative histogram (left, **I and K**) or dot plots (left, **J**) and MFI (I and K, right) or frequency (J, right). Data are given as means ± standard error of the means (SEMs). N.S.: not significant. *p < 0.05, **p < 0.01, and ***p < 0.001. p values were obtained by using a two-way analysis of variance (ANOVA) followed by Bonferroni’s post-test (**A, C, and H**) and the Student’s *t*-test (**B, D, E, and I–K**). Data were pooled from two (**B, H, and F**) or three (**A**, C–E, **G, and I–K**) independent experiments. Figure 2—source data 1.Source data for Figure 2A-K.

Previous reports have demonstrated that the number of Treg cells in melanoma is correlated with accelerated tumor growth (Mougiakakos et al., 2010). In contrast, depletion of Treg cells leads to less melanoma growth. To elucidate how CD300a on DCs enhances the adaptive immune response against tumor development, we analyzed the population of tumor-infiltrating Treg cells by use of flow cytometry and immunohistochemistry. The Treg cell population was larger in the tumor, but not the draining lymph nodes, of *Cd300a^−/−^* mice compared with that of wild-type mice (Figure 2A and B). Likewise, *Cd300a*^fl/fl^;*Itgax*^Cre^ mice showed a higher number of tumor-infiltrating Treg cells than did *Cd300a*^fl/fl^ mice (Figure 2C). These Treg cells in *Cd300a*^fl/fl^;*Itgax*^Cre^ mice showed higher expression of Ki67 and CTLA-4 than did *Cd300a*^fl/fl^ mice, although the expressions of CD25, GITR, and BCL2 on tumor-infiltrating Treg cells were comparable between two genotypes of mice (Figure 2D and E, Figure 2—figure supplement 1).

**Figure 2—figure supplement 1.**
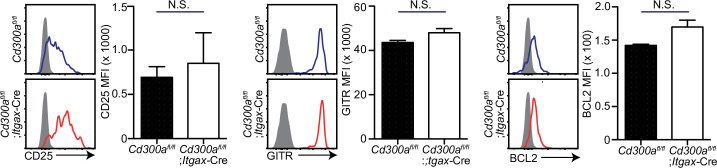
Expression of CD25, GITR, and BCL2 in tumor-infiltrating Treg cells is independent of CD300a. Flow cytometric analysis of the expressions of CD25, GITR, and BCL2 in Treg cells in the tumor in *Cd300a^fl/fl^* (*n* = 3) and *Cd300a*^fl/fl^;*Itgax*^Cre^ mice (*n* = 4). Representative histogram (left) and mean fluorescent intensity (MFI, right). Data are given as means ± standard error of the means (SEMs). N.S.: not significant. Figure 2—figure supplement 1—source data 1.Source data for Figure 2—figure supplement 1.

**Figure 2—figure supplement 2.**
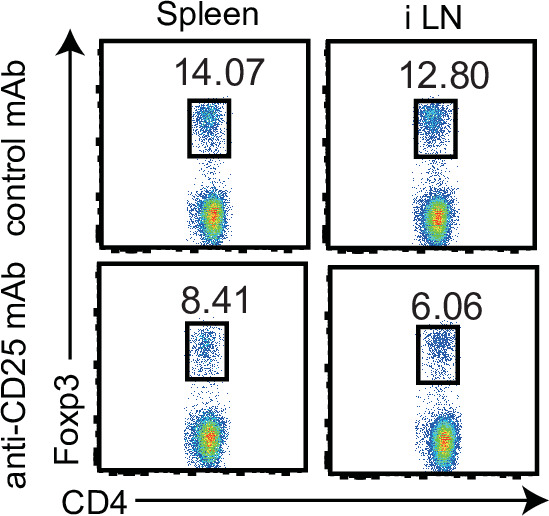
Anti-CD25 mAb reduces Treg cells. Flow cytometric analysis of Foxp3^+^ cells in the spleen and inguinal lymph node (iLN) of mice injected with isotype mAb and 300 μg of anti-CD25 mAb on days −6 and −3 prior to analysis. Data are representative of two independent experiments with similar results. Data are representative of three mice.

To determine whether Treg cells were indeed involved in the exacerbated tumor growth of *Cd300a^−/−^* mice, we depleted Treg cells by using an anti-CD25 monoclonal antibody (mAb) (Onizuka et al., 1999; Figure 2—figure supplement 2). After Treg cell depletion, the tumor volume of the *Cd300a^−/−^* and *Cd300a*^fl/fl^;*Itgax*^Cre^ mice decreased to a level comparable to that seen in wild-type and *Cd300a*^fl/fl^ mice, respectively (Figure 2F and G). Tumor-infiltrating CD8^+^ T cells in *Cd300a^−/−^* mice expressed significantly less IFN-γ and significantly higher PD-1 than did those in wild-type mice (Figure 2H and I). Moreover, the number of CD8^+^ T cells expressing both PD-1 and TIM-3 and those expressing CTLA-4 were significantly increased in *Cd300a*^fl/fl^;*Itgax*^Cre^ mice compared to *Cd300a*^fl/fl^ mice (Figure 2J and K), suggesting that tumor-infiltrating CD8^+^ T cells display more exhausted state in *Cd300a*^fl/fl^;*Itgax*^Cre^ mice than in *Cd300a*^fl/fl^ mice (Sawant et al., 2019). These results suggest that CD300a on DCs regulates the number of tumor-infiltrating Treg cells, which suppress tumor immune responses.

### TEVs augment IFN-β production by DCs

**Figure 3.**
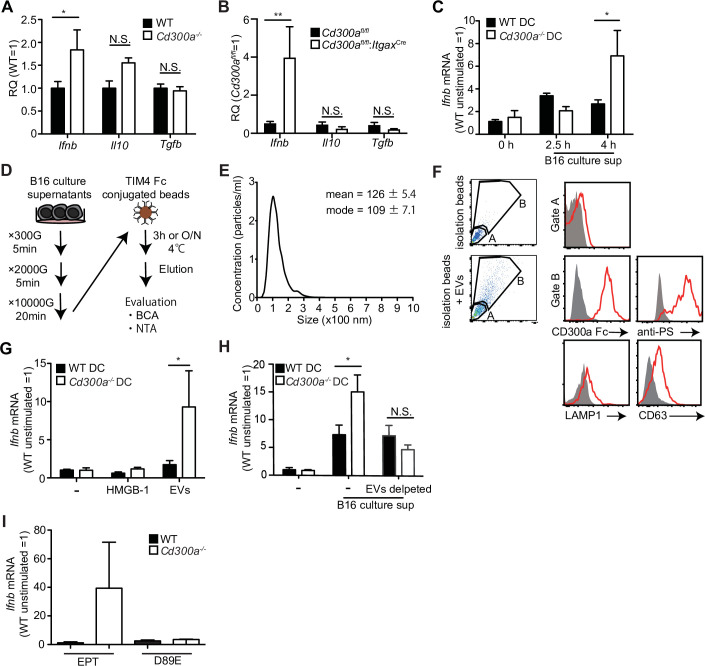
Tumor-derived extracellular vesicles (EVs) facilitate interferon-β (IFN-β) production from dendritic cells. (**A and B**) Quantitative everse transcription PCR (RT-PCR) analysis of mRNA from CD11c+ cells sorted from B16 melanoma in wild-type (WT), Cd300afl/fl (n = 6), Cd300a−/− or Cd300afl/fl;ItgaxCre (n = 6) mice 2 weeks after tumor inoculation. Results are presented relative to those of the control gene encoding β-actin. (**C**) Quantitative RT-PCR analysis of Ifnb in WT- and Cd300a−/−-derived bone marrow-derived dendritic cells (BMDCs) that received no treatment (0 hr, n = 7) or B16 culture supernatants (2.5 hr, n = 5; 4.0 hr, n = 7). (**D**) A schematic illustration of EV isolation. (**E**) The size distribution of isolated B16-derived EVs was analyzed by NTA using NanoSight LM10. (**F**) Flow cytometric analysis of EVs isolated from B16 melanoma supernatants. Bead-conjugated EVs were analyzed by flow cytometry and characterized by the indicated antibody in the presence of 2 mM CaCl_2_. (**G and H**) Quantitative RT-PCR analysis of Ifnb in WT and Cd300a^−/−^ BMDCs that received no treatment (–) (n = 6 in each group) and were treated with high-mobility group Box 1 protein (HMGB-1) (n = 3 in each group) or B16-derived EVs (n = 5 in each group) (**G**) or cocultured with B16 cultured supernatant with or without the depletion of EVs (n = 5 in each group) (**H**). (**I**) Quantitative RT-PCR analysis of Ifnb in WT and Cd300a^−/−^ BMDCs that were treated with EPT (control protein; EPT-MFG-E8, n = 3 in each group) or D89E (D89E-MFG-E8, n = 3 in each group). Data are given as means ± standard error of the means (SEMs). RQ: relative quantification; N.S.: not significant. *p < 0.05 and **p < 0.01. p values were obtained by using a two-way analysis of variance (ANOVA) followed by Bonferroni’s post-test (**A–C, G, and H**). Data were pooled from three (**A–C, G, and H**) independent experiments. Figure 3—source data 1.Source data for Figure 3A-C and G-I.

We previously reported that a microbiota-mediated signal induces increased IFN-β production by DCs and increased numbers of Treg cells in the barrier tissues such as the intestine, skin, and airway of *Cd300a^−/−^* mice relative to those of wild-type mice (Nakahashi-Oda et al., 2016). In the current study, we found that the expression of *Ifnb* was also higher in DCs in the tumor tissues of *Cd300a^−/−^* and *Cd300a*^fl/fl^;*Itgax*^Cre^ mice than in those of wild-type and *Cd300a*^fl/fl^ mice, resepectively (Figure 3A and B). To examine whether the microbiota is also involved in Treg cell levels in the tumor and tumor growth, we used wild-type and *Cd300a^−/−^* mice raised under the germ-free (GF) conditions. In contrast to the barrier tissues, *Cd300a^−/−^* mice still showed larger numbers of Treg cells and a larger tumor volume than did wild-type mice raised under GF conditions (Figure 3—figure supplement 1). These results suggest that, unlike in the barrier tissues, the microbiota-mediated signal was dispensable for the increased numbers of Treg cells in the tumor and for the enhanced tumor growth in *Cd300a^−/−^* mice.

**Figure 3—figure supplement 1.**
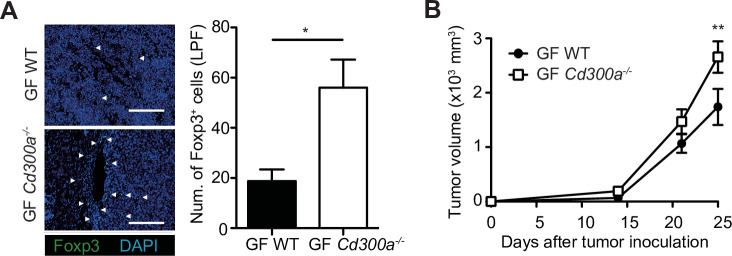
Tumor growth in *Cd300a^−/−^* mice is independent of the microbiota. (**A**) Representative fluorescence micrographs of tumor sections from germ-free (GF) wild-type (WT) and *Cd300a^−/−^* mice and stained with an anti-Foxp3 monoclonal antibody (green) and the DNA-binding dye 4′,6-diamidino-2-phenylindole (DAPI; left). The number of Foxp3^+^ cells was quantified from four high-power fields (LPF) (right). White arrows show Foxp3-positive cells. Scale bar, 200 μm. (**B**) Comparison of tumor growth of B16 melanoma between GF WT (*n* = 5) and *Cd300a^−/−^* mice (*n* = 6). Data are given as means ± standard error of the means (SEMs). *p < 0.05 and **p < 0.01. p values were obtained by using the Student’s *t*-test (**A**) and a two-way analysis of variance (ANOVA) followed by Bonferroni’s post-test (**B**). Data were pooled from two independent experiments (**A and B**). Figure 3—figure supplement 1—source data 1.Source data for Figure 3—figure supplement 1.

Solid tumors lapse into necrosis in the core region under conditions of hypoxia and low pH, resulting in the secretion of several immune stimulators, such as damage-associated molecular patterns (DAMPs), DNA, RNA (Patidar et al., 2018), and EVs (Couto et al., 2018). We examined whether the culture supernatant of B16 melanoma cells containing tumor-derived immune mediators had any effect on *Ifnb* expression by using cultured bone marrow-derived dendritic cells (BMDCs). Four hours after incubation in the presence of the culture supernatant, *Cd300a^−/−^* BMDCs expressed higher levels of *Ifnb* than did wild-type BMDCs (Figure 3C), suggesting that CD300a suppressed the *Ifnb* expression induced by a tumor-derived immune mediator in the culture supernatant. Since EVs are the particles released from the cells that are delimited by a lipid bilayer that contains phosphatidylserine (Lima et al., 2009), the ligand for CD300a (Nakahashi-Oda et al., 2012a), and containing functional biomolecules (van Niel et al., 2018). We purified TEVs from the culture supernatants of B16 melanoma cells by centrifugation and phosphatidylserine receptor-conjugated beads (Figure 3D and E), which indeed expressed phosphatidylserine on the surface and bound to a chimeric fusion protein of the extracellular portion of CD300a with human IgG1 (Figure 3F). Stimulation with the purified TEVs induced higher *Ifnb* expression in *Cd300a^−/−^* BMDCs than in wild-type BMDCs (Figure 3G). However, *Cd300a^−/−^* BMDCs showed decreased *Ifnb* expression to a level comparable to wild-type BMDC when these BMMCs were cultured in the culture supernatants of B16 melanoma cells after removal of TEVs by phosphatidylserine receptor-conjugated beads (Figure 3H). In contrast, neither wild-type nor *Cd300a^−/−^* BMDCs expressed IFN-β after stimulation high-mobility group box-1 protein (HMGB-1) (Figure 3G) a well-known DAMP, which can be released by damaged tumors. These results suggest that the interaction of CD300a with PS on TEVs suppressed *Ifnb* expression in BMDCs. Indeed, *Ifnb* expression in *Cd300a^−/−^* BMDCs was decreased to a level comparable to that seen in wild-type BMDCs after stimulation with TEVs whose PS was masked with MFG-E8 protein mutated at residue 89 (D89E-MFG-E8) (Nakahashi-Oda et al., 2012a; Figure 3I). Together, these results indicated that TEVs suppressed *Ifnb* expression in BMDCs via interaction between CD300a on BMDCs and PS on TEVs.

### TEVs enhanced Treg cell proliferation and consequent tumor development via IFN-β

**Figure 4.**
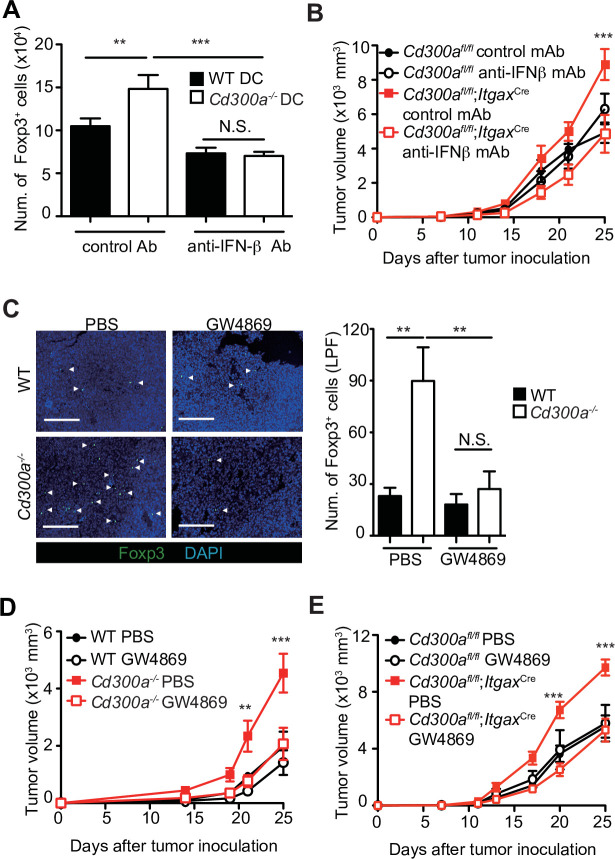
Tumor-derived extracellular vesicles (TEVs) promote tumor-infiltrating Treg cell accumulation. (**A**) The number of induced Foxp3-eGFP^+^ cells (iTreg) generated from naive T cells by using anti-CD3, anti-CD28, Interleukin-2 (IL-2), and Transforming growth factor-β (TGF-β). These iTreg cells were cocultured with TEV-stimulated bone marrow-derived dendritic cells (BMDCs) in the presence of IL-2 and TGF-β for 5 days with a control mAb (*n* = 7) or an anti-interferon-β (IFN-β) mAb (*n* = 5). (**B**) Tumor growth curves of *Cd300a^fl/fl^* and *Cd300a*^fl/fl^;*Itgax*^Cre^ mice treated with an anti-IFN-β mAb or a control mAb (control, *n* = 7; anti-IFN-β, *n* = 7 for each genotype mouse) three times (days 7, 11, and 14) and after inoculation of B16 melanoma cells. (**C**) Representative fluorescence micrographs of tumor sections from Foxp3-eGFP wild-type (WT) (phosphate-buffered saline (PBS), *n* = 4; GW4869, *n* = 6) and Foxp3-eGFP *Cd300a^−/−-^* mice (PBS, *n* = 5; GW4869, *n* = 6) in the absence or presence of GW4869, and stained with an anti-GFP mAb (green) and the DNA-binding dye 4′,6-diamidino-2-phenylindole (DAPI, left). The number of Foxp3^+^ cells was quantified from four high-power fields (LPF) (right). White arrow shows Foxp3-positive cells. Scale bar, 200 μm. Tumor growth curves of WT (PBS, *n* = 6; GW4869, *n* = 9) (**D**) or *Cd300a*^fl/fl^ (*n* = 4 each) (**E**) and *Cd300a^−/−^* mice (PBS, *n* = 7; GW4869, *n* = 9) (**D**) or *Cd300a*^fl/fl^;*Itgax*-Cre (*n* = 6 each) (**E**) that were treated with GW4869 or PBS three times (days 14, 18, and 21). Data are given as means ± standard error of the means (SEMs). RQ: relative quantification; N.S.: not significant. **p < 0.01 and ***p < 0.001. p values were obtained by using a one-way analysis of variance (ANOVA) (**A and C**) and a two-way ANOVA followed by Bonferroni’s post-test (**B**, **D, and E**). Data were pooled from two (**A and C**) or three (**B, D, and E**) independent experiments. Figure 4—source data 1.Source data for Figure 4A-E.

To clarify whether IFN-β enhances Treg cell proliferation, we cocultured TEV-stimulated wild-type or *Cd300a^−/−^* BMDCs with Treg cells that were generated from naive CD4^+^ T cells from Foxp3-eGFP^+^ mice in the presence of anti-CD3 and anti-CD28 mAbs, IL-2, and TGF-β. TEV-stimulated *Cd300a^−/−^* BMDCs increased the number of Treg cells to a greater extent than did TEV-stimulated wild-type BMDCs (Figure 4A). However, TEV-stimulated *Cd300a^−/−^* BMDCs cocultured with Foxp3-eGFP^+^ Treg cells rather than naïve CD4^+^ T cells had the same number of Treg cells as when cocultured with wild-type BMDCs (Figure 4—figure supplement 1). Addition of a neutralizing anti-IFN-β antibody to the coculture of Treg cells and *Cd300a^−/−^* BMDCs reduced the Treg cell numbers to a level comparable to that seen in the coculture of Treg cells and wild-type BMDCs (Figure 4A). Moreover, administration of a neutralizing anti-IFN-β antibody showed reduced tumor volume in *Cd300a*^fl/fl^;*Itgax*^Cre^ mice to a comparable level of that in *Cd300a*^fl/fl^ mice (Figure 4B), suggesting that IFN-β augmented Treg cell proliferation or survival and promoted tumor progression in *Cd300a*^fl/fl^;*Itgax*^Cre^ mice. To investigate the effects of TEVs on Treg cells, we injected an EV-release inhibitor GW4869 (Ikebuchi et al., 2018) into the tumor region on days 10, 14, and 18 after tumor inoculation. Treatment with GW4869, which inhibits EV release from tumors and DCs, led to a significant decrease in the number of tumor-infiltrating Treg cells and the tumor volume in *Cd300a^−/−^* and *Cd300a*^fl/fl^;*Itgax*^Cre^ mice to a comparable level of those in wild-type and *Cd300a*^fl/fl^ mice, respectively (Figure 4C-E). These results nevertheless indicate that CD300a suppresses TEV-mediated IFN-β production, resulting in a decrease in the Treg cell population and the suppression of tumor development.

**Figure 4—figure supplement 1.**
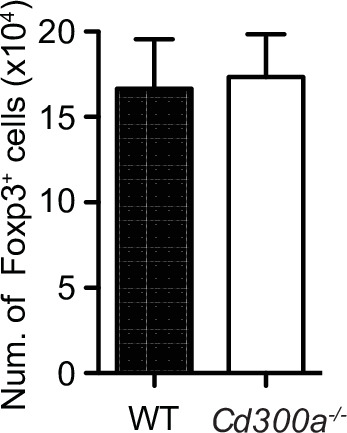
CD300a suppression of bone marrow-derived dendritic cells (BMDCs) does not affect the number of Foxp3^+^ splenic regulatory T (Treg) cells. Flow cytometric data of the number of Treg cells. Foxp3-eGFP^+^ cells (nTreg) were isolated from spleen and cocultured with extracellular vesicle (EV)-stimulated BMDCs from wild-type (WT) and *Cd300a^−/−^* for 5 days. Figure 4—figure supplement 1—source data 1.Source data for Figure 4—figure supplement 1.

### CD300a inhibits the EV-induced TLR3–TRIF signaling for IFN-β production

**Figure 5.**
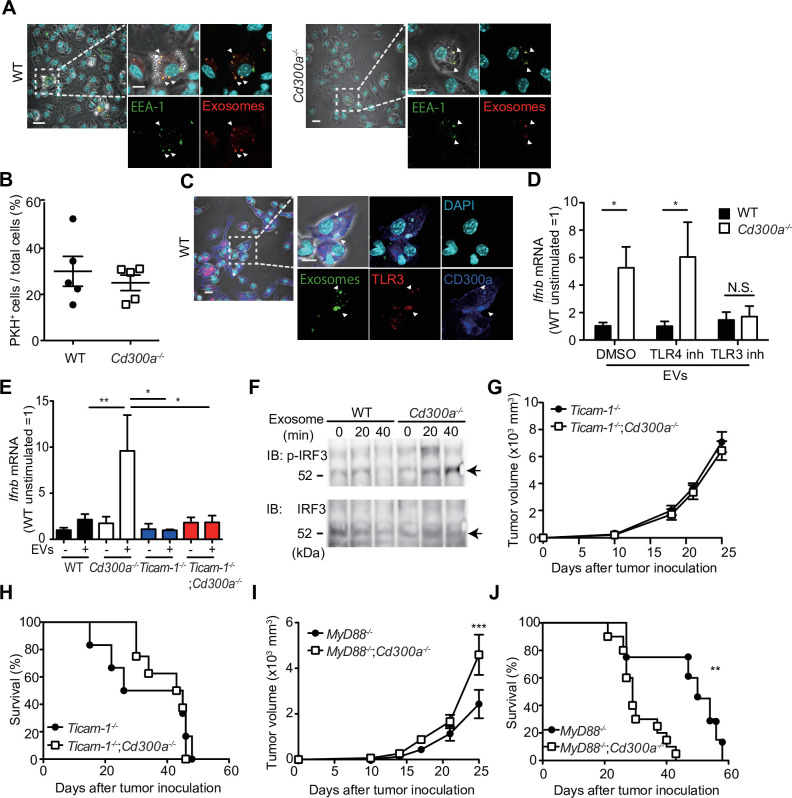
CD300a inhibits TLR3-mediated interferon-β (IFN-β) expression upon recognition of tumor-derived exosomes. (**A**) Representative microscopy images of wild-type (WT) and *Cd300a^−/−^* bone marrow-derived dendritic cells (BMDCs) treated with pHrodo-labeled extracellular vesicles (EVs) to assess the localization of EVs (red) and early endosome antigen (EEA)-1 (green). Scale bar, 10 μm. Data are representative of two independent experiments. (**B**) Uptake of PKH-labeled tumor-derived EVs (TEVs) in WT (*n* = 5) and *Cd300a^−/−^* BMDCs (*n* = 5). (**C**) Representative microscopy images of WT and *Cd300a^−/−^* BMDCs treated with pHrodo-labeled exosomes to assess the localization of exosomes (green), TLR3 (red), and CD300a (blue). Scale bar, 10 μm. Data are representative of two independent experiments. (**D**) Quantitative RT-PCR analysis of *Ifnb* in WT and *Cd300a^−/−^* BMDCs treated with B16-derived exosomes in the presence of dimethyl sulfoxide (DMSO) (WT, *n* = 9; *Cd300a^−/−^*, *n* = 10), 100 nM TLR4 inhibitor (*n* = 7 in each group), and 50 μM TLR3 inhibitor (*n* = 6 in each group). (**E**) Quantitative RT-PCR analysis of *Ifnb* in WT, *Cd300a^−/−^*, *ticam-1^−/−^*, and *ticam-1^−/−^;Cd300a^−/−^* mice-derived BMDCs treated with B16-derived EVs (*n* = 5 in all group). (**F**) Representative immunoassay of WT and *Cd300a^−/−^* BMDCs left unstimulated (0 min) or stimulated for the indicated times with B16-derived exosomes, followed by immunoblot analysis of phosphorylated (p-) interferon regulatory factor 3 (IRF3) or total IRF3. Data are representative of two independent experiments. (**G and H**) Comparison of tumor growth and survival curves of B16 melanoma cells between *ticam-1^−/−^* (*n* = 6) and *ticam-1^−/−^;Cd300a^−/−^* ice (*n* = 9) after inoculation of B16 melanoma. (**I and J**) Comparison of tumor growth and survival curves of B16 melanoma between *MyD8^−/−^* (*n* = 9) and *MyD88^−/−^;Cd300a^−/−^* mice (*n* = 10) after inoculation of B16 melanoma. Data are given as means ± standard error of the means (SEMs). N.S.: not significant. *p < 0.05, **p < 0.01, and ***p < 0.001. p values were obtained by using the Student’s *t*-test (**B**), a two-way analysis of variance (ANOVA) followed by Bonferroni’s post-test (**D, E, G, and I**), and the log-rank test (**H and J**). Data were pooled from two (**B, E, and H**) or three (**D, I, and J**) independent experiments. Figure 5—source data 1.Source data for Figure 5B, D, E, and G-J.

To further analyze how CD300a regulates TEV-mediated IFN-β production in DCs, we cocultured pHrodo- or PKH-labeled exosomes with wild-type or *Cd300a^−/−^* BMDCs and analyzed the localization of the TEVs in BMDCs by using confocal laser scanning microscopy. We found that the TEVs were incorporated into endosomes, as identified by the expression of endosome antigen (EEA)-1, in both genotypes of DCs (Figure 5A). The number of TEVs in the endosomes was comparable between wild-type and *Cd300a^−/−^* BMDCs (Figure 5B), suggesting that CD300a did not affect TEV incorporation into the endosomes. Interestingly, we also found that CD300a was internalized from the cell surface into the endosomes, an event that might be mediated by the tyrosine-based sorting motif in the cytoplasmic region of CD300a (Yotsumoto et al., 2003), after coculture of BMDCs with TEVs (Figure 5C and Figure 5—figure supplement 1). As a result, the TEVs colocalized with CD300a at the endosomes (Figure 5A and C). Given that EVs expose phosphatidylserine on their lipid bilayer, which is a CD300a ligand, these results suggest that CD300a was activated via stimulation with TEVs at the endosomes.

**Figure 5—figure supplement 1.**
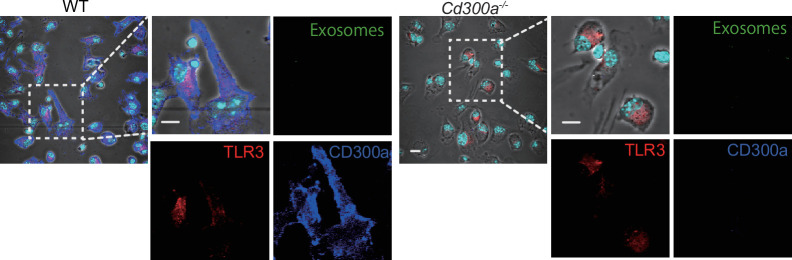
CD300a is localized on the surface of plasma membrane without stimulation. Representative confocal microscopy images of bone marrow-derived dendritic cells (BMDCs) stained with anti-TLR3 and anti-CD300a mAbs. Data were pooled from two independent experiments.

**Figure 5—figure supplement 2.**
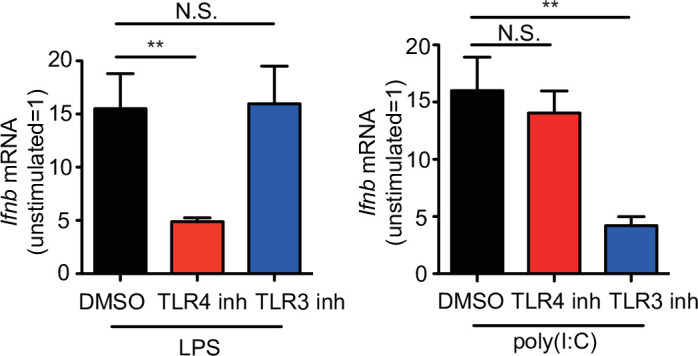
The inhibitors of TLR4 and TLR3 suppress the expression of *Ifnb*. Quantitative RT-PCR analysis of *Ifnb* in bone marrow-derived dendritic cells (BMDCs) stimulated with LPS or poly(I:C) in the presence of TLR4 or TLR3 inhibitors (LPS and poly(I:C), *n* = 12 in each group; TLR4 and TLR3 inhibitors, *n* = 9 in each group). Data are given as means ± standard error of the means (SEMs). N.S.: not significant. **p < 0.01. p values were obtained by using a one-way analysis of variance (ANOVA) followed by Bonferroni’s post-test. Data were pooled from three independent experiments. Figure 5—figure supplement 2—source data 1.Source data for Figure 5—figure supplement 2.

EVs also contain nucleic acids, including structured RNA (Liu et al., 2016; van Niel et al., 2018). TLR3 at the endosomal membrane can recognize RNA and mediates IFN-β production via the TRIF signaling pathway in DCs (Tatematsu et al., 2013). To examine whether CD300a inhibited TLR3-mediated signaling at the endosomes upon stimulation with TEVs, we cocultured wild-type and *Cd300a^−/−^* BMDCs with TEVs in the presence of an inhibitor of TLR3 (Cheng et al., 2011). This inhibitor decreased *Ifnb* expression in *Cd300a^−/−^* BMDCs to a level comparable to that in wild-type BMDCs (Figure 5D and Figure 5—figure supplement 2). In contrast, the TLR4 inhibitor TKA-242 did not affect the expression of *Ifnb* in either BMDC genotype (Figure 5D and Figure 5—figure supplement 2). These results suggest that CD300a inhibits TLR3-mediated signaling for IFN-β production. Moreover, the expression of *Ifnb* in *ticam-1^−/−^;Cd300a^−/−^* BMDCs was also decreased to the comparable level of that in *ticam-1^−/−^* BMDCs after coculture with TEVs (Figure 5E). In addition, we found that the phosphorylation level of interferon regulatory factor 3 (IRF3), a downstream molecule of the TRIF signaling pathway, was increased to a greater extent in EV-stimulated *Cd300a^−/−^* BMDCs than in wild-type BMDCs (Figure 5F). In vivo analyses also showed that, although tumor growth was significantly larger and the survival rate was significantly shorter for B16 melanoma-injected *Myd88^−/−^;Cd300a^−/−^* mice compared with B16-injected *Myd88^−/−^* mice, tumor development and survival did not differ between *ticam-1^−/−^;Cd300a^−/−^* and *ticam-1^−/−^* mice (Figure 5G-J). Taken together, these data suggest that CD300a inhibits the TLR3–TRIF signaling pathway for IFN-β production at the endosomes in DCs, resulting in the suppression of Treg cell activation and tumor development.

### *CD300A* expression associates with survival times in melanoma patients

**Figure 6.**
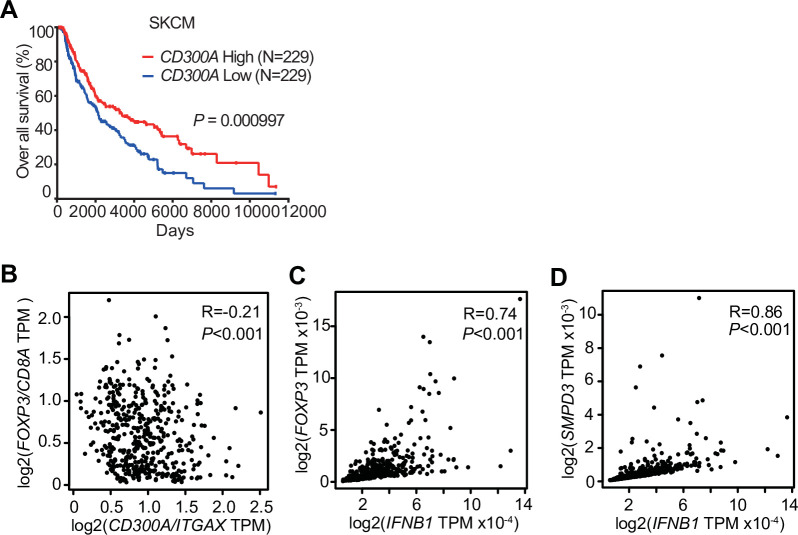
*CD300A* expression associates with survival of human melanoma patients. (**A**) Kaplan plot showing low and high *CD300A* expressions in skin cutaneous melanoma (SKCM) patients obtained by performing a meta-analysis of The Cancer Genome Atlas (TCGA) database. Median values were used as thresholds (numbers of both low and high expression patients = 229). (**B–D**) Spearman correlation analysis of TCGA skin cutaneous melanoma database by using GEPIA2. *FOXP3*, *IFNB1*, and *SMPD3* expression were normalized by *GAPDH* expression (**C and D**).

To examine the role of CD300A in tumor development in humans, we analyzed the data on the single-cell RNA sequence (scRNA-seq) of human melanoma tissues, which demonstrated that *CD300A* is expressed on populations that express *HLA-DR*, *ITGAX (CD11C)*, *ITGAM (CD11B)*, *CD14*, and *CD163* (Figure 6—figure supplement 1), consistent with the results of mouse melanoma. We further analyzed the database of the Cancer Genome Atlas (TCGA) project and found that skin cutaneous melanoma (SKCM) patients expressing low levels of *CD300A* mRNA had shorter survival times than did those expressing higher *CD300A* mRNA levels (Figure 6A). We also found that the expression ratio of *CD300A* to *ITGAX* is negatively correlated with that of *FOXP3* to *CD8A* (Figure 6B). These results suggested that CD300A suppressed Treg cell proliferation and/or activation and tumor development. Moreover, we found that patients with melanoma showed strong positive correlation between *FOXP3* and *IFNB1* expression (Figure 6C). Neutral sphingomyelinase-2 (SMPD3), which is a target of an inhibitor of EV-release GW4869, enhances TEV release from tumor cells (Kosaka et al., 2013; Kosaka et al., 2010). TCGA database of SKCM also showed a strong positive correlation between expressions of *SMPD3* and *IFNB1* in melanoma tissues (Figure 6D), suggesting that TEVs increased IFN-β expression in human melanoma tissues. These results were consistent with those of mouse models of melanoma development in the current study. Taken together, these results suggested that CD300A might augment tumor immunity via suppression of tumor-infiltrating Treg cells also in humans.

**Figure 6—figure supplement 1.**
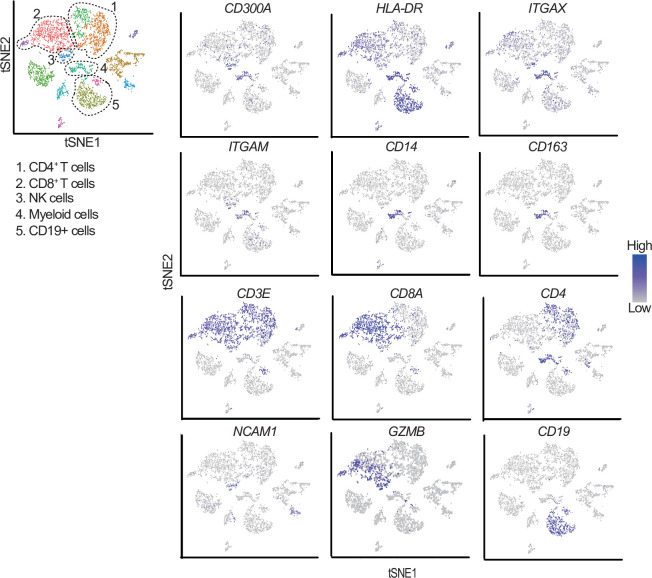
*t*-Distributed stochastic neighbor embedding (tSNE) plots of the immune cell landscape isolated from melanoma patients. Indicated gene expressions were reanalyzed by using GSE76056. Cell clusters of CD4^+^ T cells, CD8^+^ T cells, NK cells, myeloid cells, and CD19^+^ cells were determined by *CD4/CD3E*, *CD8A/CD3E*, *NCAM1(CD56)/GZMB*, *ITGAM(CD11B*)/*ITGAX(CD11C*)/HLA-DR/CD14/CD163, and *CD19* expression, respectively.

## Discussion

**Figure 7.**
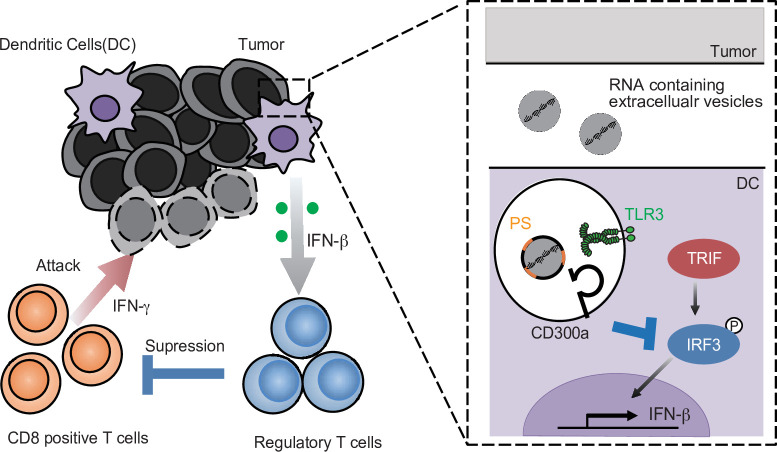
A schematic model of the role of tumor-derived extracellular vesicle (TEV) and CD300a in tumor immunity. RNA-containing TEV is incorporated into the endosomes in dendritic cells (DCs), where phosphatidylserine (PS) on TEV binds to CD300a and inhibits the TLR3–TRIF–IRF3 signaling pathway initiated by TEV-derived RNA binding to TLR3, resulting in the decrease in interferon-β (IFN-β) production by DCs and the number of tumor-infiltrating Treg cells. The Treg cells regulate tumor development.

Although the biological roles of EVs have been reported from various angles, how EVs regulate immune responses is not yet fully understood. In the present study, we showed that TEV-stimulated DCs for IFN-β production via TLR3 at the endosomes, resulting in the increased number of tumor-infiltrating Treg cells and thus the exacerbation of tumor development. In contrast, the TEVs also stimulated CD300a and inhibited TEV-mediated TLR3 signaling at the endosome. Thus, TEVs have both positive and negative functions in the regulation of IFN-β production and Treg activation via the axis of EV-derived RNA-TLR3 and EV-derived phosphatidylserine-CD300a, respectively. These results suggest that the Treg cells in tumor microenvironments is regulated by the balance of positive and negative signaling for IFN-β production induced by TEV (Figure 7). Hence, it is an interesting issue to be examined whether the expressions of RNAs are different among TEVs derived from tumors of variable tissue types.

On the other hand, the balance of TLR3 and CD300a expressions in DCs may also be important for Treg activation and tumor development. Indeed, we showed that higher expression of CD300A was associated with lower expression of Foxp3 and longer survival times of melanoma patients. Though human CD300A is reported to be expressed on the subsets of T cells in peripheral blood (Borrego, 2013), human melanoma tissues showed quite low CD300A expression in T cells compared to high expression in the myeloid cells. Further analysis of human CD300A expressions on T cells in tumor microenvironment is needed. While previous reports demonstrated that TEVs promoted Treg cell expansion through DC-independent manner in vitro (Muller et al., 2017; Szajnik et al., 2010; Wieckowski et al., 2009), the current study first demonstrated that TEVs regulate Treg cell activation and tumor development in vivo by DCs in the tumor microenvironment. Meanwhile, Tumor-infiltrating DCs are heterogenous, and can be divided into at least two subsets. The conventional type-1 DC (cDC1) expresses the chemokine receptor XCR1 and CD103 and lower amount of CD11b that has the high ability to migrate from tumors to lymph nodes and presents a tumor antigen to CD8^+^ T cells (Bedoui et al., 2009). In contrast, the conventional type-2 DC (cDC2) are commonly distinguished from cDC1 by their preferential expression of higher amount of CD11b. cDC2 are predominantly involved in antigen presentation by MHC class II to CD4^+^ T cells (Gao et al., 2013). Given that cDC2 is involved in CD4^+^ T cell differentiation and activation, CD300a on cDC2, rather than cDC1, may regulate Treg cells activation by inhibiting the TLR3–IFN-β pathway in tumor microenvironment.

Type I IFNs are key players in antiviral and anticancer immune response by upregulating both cross-presentation of antigens by CD8a^+^ DCs and cytotoxic activity of CD8^+^ T cells and NK cells (Zitvogel et al., 2015). However, the current clinical use of IFN-β for cancers showed limited efficiency (Medrano et al., 2017; Minn, 2015). This may be partly due to the fact that type I IFNs also have immunosuppressive functions, such as inducing the production of IL-10, an immunosuppressive cytokine (Snell et al., 2017). In addition, on Treg cells, IFNs are most potent cytokines to induce PD-L1 (Morimoto et al., 2018; Xiao et al., 2018), which contributes to sustain Foxp3 expression and promotes the function of Treg cells (Francisco et al., 2009). In addition, IFN-α/β receptor signaling promotes Treg cell development (Metidji et al., 2015). We previously reported that gut commensals stimulated CX3CR1^+^CD103^−^ CD11b^+^ DCs to produce IFN-β, which augmented the proliferation of Treg cells in the intestine (Nakahashi-Oda et al., 2016). In contrast, published reports demonstrated that, in viral infection and tumor microenvironment, type I IFNs directly inhibit the proliferation and activation of Treg cells (Gangaplara et al., 2018; Srivastava et al., 2014). Further investigations are required to clarify whether such IFN-β-induced Treg cell increase is caused by Treg cell-intrinsic or Treg cell-extrinsic effect by the cytokine in the tumor microenvironment.

We have previously reported that CD300a inhibited the CD14-mediated TLR4 internalization in CD11b^+^ DCs induced by gut microbiota (Nakahashi-Oda et al., 2016). This internalization of TLR4 by CD14 induces activation of the TRIF pathway to produce IFN-β, but not MyD88 pathway. In the present study, we also found that CD300a inhibits TLR3-mediated TRIF signaling to produce IFN-β (Figure 5D and E). These data suggest that CD300a specifically inhibits TRIF signaling to produce IFN-β, rather than the MyD88-mediated signaling pathway stimulated by LPS and HMGB-1. TLR3 activates PI3 kinase and the downstream kinase, Akt, leading to full phosphorylation and activation of IRF3 (Sarkar et al., 2004). Indeed, we showed that IRF3 phosphorylation was increased in *Cd300a^−/−^* DCs compared with wild-type DCs after stimulation with TEV. Recent studies have revealed that TLR3 on alveolar epithelial cells recognized RNAs in TEV and promoted lung metastasis (Liu et al., 2016). Therefore, the role of RNAs in TEV is dependent on target cells. Our findings thus highlighted the role of TEV and CD300a on DCs in the regulation of tumor-infiltrating Treg cells and tumor immunity.

## Materials and methods

**Key resources table:** 

Reagent type (species) or resource	Designation	Source or reference	Identifiers	Additional information
Genetic reagent (*M. musculus*)	*Cd300a^−/−^*	PMID:26855029		
Genetic reagent (*M. musculus*)	*Cd300a* ^fl/fl^	PMID:26855029		
Genetic reagent (*M. musculus*)	*Cd300a*^fl/fl^;*Lyz2*^Cre^	PMID:26855029		
Genetic reagent (*M. musculus*)	*Cd300a*^fl/fl^;*Itgax*^Cre^	PMID:26855029		
Genetic reagent (*M. musculus*)	*Ticam1^−/−^;Cd300a^−/−^*	PMID:26855029		
Genetic reagent (*M. musculus*)	*MyD88^−/−^;Cd300a^−/−^*	PMID:26855029		
Genetic reagent (*M. musculus*)	*Rag1^−/−^;Cd300a^−/−^*	PMID:26855029		
Genetic reagent (*M. musculus*)	*Ticam1^−/−^*	PMID:1285581 Oriental Bio Service		
Genetic reagent (*M. musculus*)	*MyD88^−/−^*	PMID:9697844Oriental Bio Service		
Genetic reagent (*M. musculus*)	*Rag1^−/−^*	Jackson Laboratory		
Genetic reagent (*M. musculus*)	*Foxp3* ^eGFP^	PMID:18209052		Dr. B. Malissen (UM2 Aix-Marseille Université)
Genetic reagent (*M. musculus*)	*Foxp3* ^eGFP^ *;Cd300a^−/−^*	PMID:26855029		
Cell line (*M. musculus*)	B16	RIKEN Cell Bank	RCB1283RRID:CVCL_ F936	
Antibody	Rat anti-CD8-PE-Cy7 (53–6.7, mouse monoclonal)	BD Bioscience	Cat# 552,877RRID:AB_ 394,506	FACS (1:5)
Antibody	Rat anti-CD4-APC (RM4-5, mouse monoclonal)	BD Bioscience	Cat# 553,051RRID:AB_398528	FACS (1:5)
Antibody	Rat anti-CD11b-APC-Cy7 (M1/70, monoclonal)	BD Bioscience	Cat# 557,657RRID:AB_396772	FACS (1:5)
Antibody	Armenian hamster anti-CD11c FITC (HL3, monoclonal)	BD Bioscience	Cat# 553,801RRID:AB_553801	FACS (1:5)
Antibody	Rat anti-I-A/I-E BV500 (M5/114.15.2, monoclonal)	BD Bioscience	Cat# 562,366RRID:AB_11153488	FACS (1:5)
Antibody	Rat anti-Ly6G PE (1A8, monoclonal)	BD Bioscience	Cat# 551,461RRID:AB_394208	FACS (1:5)
Antibody	Rat anti-CD62L PE (MEL-14, monoclonal)	BD Bioscience	Cat# 553151, RRID:AB_394666	FACS (1:5)
Antibody	Rat anti-CD44 APC (IM7, monoclonal)	BD Bioscience	Cat# 559250, RRID:AB_398661	FACS (1:5)
Antibody	Rat anti-CD25 PE (PC61, monoclonal)	BD Bioscience	Cat# 553866, RRID:AB_395101	FACS (1:5)
Antibody	Mouse anti-Ki67 Alexa Fluor 647(B56, monoclonal)	BD Bioscience	Cat# 558615, RRID:AB_647130	FACS (1:5)
Antibody	Rat anti-IFN-g Alexa Fluor 488 (XMG1.2, monoclonal)	BD Bioscience	Cat# 557724, RRID:AB_396832	FACS (1:5)
Antibody	Rat anti-CD63 APC-Cy7 (NVG-2, monoclonal)	BioLegend	Cat# 143907, RRID:AB_2565497	FACS (1:5)
Antibody	Armenian hamster anti-CD103 APC (2E7, monoclonal)	BioLegend	Cat# 121,414RRID:AB_1227502	FACS (1:5)
Antibody	Mouse anti-XCR1 PE (ZET, monoclonal)	BioLegend	Cat# 148,204RRID:AB_2563843	FACS (1:5)
Antibody	Mouse anti-Bcl-2 PE (BCL/10C4, monoclonal)	BioLegend	Cat# 633508, RRID:AB_2290367	FACS (1:5)
Antibody	Rat anti-GITR PE-Cy7 (DTA-1, monoclonal)	BioLegend	Cat# 126317, RRID:AB_2563385	FACS (1:5)
Antibody	Rat anti-PD-1 PE-Cy7 (RMP1-30, monoclonal)	BioLegend	Cat# 109,109RRID:AB_572016	FACS (1:5)
Antibody	Rat anti-Tim3 PE (RMT3-23, monoclonal)	BioLegend	Cat# 119703, RRID:AB_345377	FACS (1:5)
Antibody	Armenian hamster anti-CTLA-4 biotin (UC10-4B9, monoclonal)	BioLegend	Cat# 106303, RRID:AB_313252	FACS (1:5)
Antibody	Mouse anti-Foxp3 Alexa Fluor 488 (150D, monoclonal)	BioLegend	Cat# 320012, RRID:AB_439748	FACS (1:5)
Antibody	Mouse anti-CD45.2 APC (104, monoclonal)	BioLegend	Cat# 109814, RRID:AB_389211	FACS (1:5)
Antibody	Rat anti-CD40 APC (3/23, monoclonal)	BioLegend	Cat# 124612, RRID:AB_1134072	FACS (1:5)
Antibody	Rat anti-IFNβ (7F-D3, monoclonal)	Yamasa	Cat# 7,891	Vivo(50 μg/mouse, three times)
Antibody	Mouse anti-PS FITC (1H6, monoclonal)	Merck Millipore	Cat# 16-256, RRID:AB_492616	FACS (1:5)
Antibody	Rat anti-CD16/CD32 (2.4G2, monoclonal)	TONBO Bioscience	Cat# 70-0161, RRID:AB_2621487	FACS (1:5)
Antibody	Mouse anti-CD300a (EX42, monoclonal)	PMID:31155312		FACS (0.1 μg)
Antibody	Rabbit anti-GFP (D5.1, monoclonal)	Cell signaling	Cat# 2956, RRID:AB_1196615	IHC(1:200)
Antibody	Rat anti-Foxp3 (FJK-16s, monoclonal)	Thermo Fisher	Cat#14-5773-82RRID:AB_467576	IHC(1:200)
Antibody	Mouse anti-EEA-1 (1G11, monoclonal)	eBioscience	Cat# 14-9114RRID:AB_2572929	ICC(1:200)
Antibody	Rat anti-TLR3 (11F8, monoclonal)	Biolegend	Cat# 141902, RRID:AB_10901162	ICC(1:200)
Antibody	Rabbit anti-phosphorylated IRF3 (4D4G, monoclonal)	Cell Signaling Technology	Cat# 4,947	WB(1:1000)
Antibody	Rabbit anti-IRF3 (FL-425, polyclonal)	Santa Cruz Biotechnology	Cat# sc-9082, RRID:AB_2264929	WB(1:1000)
Antibody	Hamster anti-CD3 purified (145–2C11, monoclonal)	TONBO Bioscience	Cat# 70-0031, RRID:AB_2621472	Cell culture(0.33 μg/ml)
Antibody	Syrian hamster anti-CD28 purified (37.51, monoclonal)	Biolegend	Cat# 102101, RRID:AB_312866	Cell culture(2 μg/ml)
Sequence-based reagent	*Ifnb*_F	This paper	PCR primers	CAGCTCCAAGAAAGGACGAAC
Sequence-based reagent	*Ifnb*_R	This paper	PCR primers	GGCAGTGTAACTCTTCTGCAT
Sequence-based reagent	*Il10*_F	This paper	PCR primers	GCTGGACAACATACTGCTAACC
Sequence-based reagent	*Il10*_R	This paper	PCR primers	ATTTCCGATAAGGCTTGGCAA
Sequence-based reagent	*Tgfb*_F	This paper	PCR primers	TGACGTCACTGGAGTTGTACGG
Sequence-based reagent	*Tgfb*_R	This paper	PCR primers	GGTTCATGTCATGGATGGTGC
Peptide, recombinant protein	Streptavidin	Thermo Fisher	Cat. #: 434,302	
Peptide, recombinant protein	GM-CSF	WAKO	Cat. #: 434,302	
Peptide, recombinant protein	IL-4	WAKO	Cat. #: 434,302	
Peptide, recombinant protein	IL-2	BD Pharmingen		
Peptide, recombinant protein	TGF-β	R&D system		
Commercial assay or kit	Exosome Isolation Kit	WAKO		
Commercial assay or kit	Tumor dissociation kit	Miltenyi Biotec		
Commercial assay or kit	High-Capacity cDNA Reverse Transcription Kit	Applied Biosystems		
Commercial assay or kit	Power SYBER Green PCR Master Mix	Applied Biosystems		
Chemical compound, drug	GW4869	Cayman Chemial	Cat. #: 13,127	
Chemical compound, drug	TLR3/dsRNA complex inhibitor	Merck	Cat. #: 614,310	
Chemical compound, drug	TLR4 inhibitor (TAK-242)	Merck	Cat. #: 614,316	
Chemical compound, drug	pHrodo Red ester	Thermo Fisher		
Chemical compound, drug	pHrodo STP Green	Thermo Fisher		
Chemical compound, drug	HRP-conjugated dextran polymer	PerkinElmer		
Software, algorithm	GraphPad Prism	GraphPad Prism		
Software, algorithm	Hybrid cell counts software	Keyence		
Software, algorithm	R Seurat	R: The R Project for Satistical Computing		
Software, algorithm	FlowJo	TreeStar		

### Mice

All gene-edited mice in the C57BL/6J background were previously described (Nakahashi-Oda et al., 2016). C57BL6J mice and GF mice were purchased from Clea Japan and Sankyo Laboratory, respectively. GF mice were bred and maintained in vinyl isolators to maintain GF conditions. Mice were used for the experiments at 8–12 weeks of age. All experiments were performed in accordance with the guidance of the animal ethics committee of the University of Tsukuba Animal Research Center.

### Antibodies, flow cytometry, and reagents

The isotype-matched control antibodies rat IgG2a (553928), rat IgG1 (553921), and mouse IgG1 (553445), as well as mAbs to CD4 (RM4-5), CD8 (53–6.7), CD11b (M1/70), CD11c (HL3), I-A/I-E (M5/114.15.2), Ly6C (AL-21), Ly6G (1A8), CD62L (MEL-14), CD44 (IM7), CD25 (PC61), Ki67 (B56), and IFN-γ (XMG1.2) were purchased from BD Bioscience. Mabs to CD63 (NVG-2), CD103 (2E7), XCR1 (ZET), Bcl-2 (BCL/10C4), GITR (DTA-1), PD-1 (RMP1-30), Tim3 (RMT3-23), CTLA-4 (UC10-4B9), Foxp3 (150D), CD45.2 (104), and CD40 (3/23) were purchased from Biolegend. Anti-IFN-β(7F-D3) was from Yamasa; control rat IgG (6130-01) was purchased from Southern Biotechnology. Anti-PS antibody (1H6) was purchased from Merck Millipore. The CD300a-specific mAb (EX42) was generated in our laboratory. Anti-CD25 (PC61) was a gift from E. Nakayama (Okayama University). Cells were treated for 10 min with anti-CD16/CD32 mAb (2.4G2; TONBO Bioscience) to prevent binding to FcγR prior to incubation with the indicated combination of antibodies. All samples were evaluated by using a Fortessa flow cytometer (Becton Dickinson) and analyzed by using FlowJo software (Tree Star).

### Tumor cell maintenance and injection

The B16 mouse melanoma cell line was obtained from RIKEN Cell Bank (Tsukuba, Japan). Authentication and mycoplasma contamination test (DNA staining method, polymerase chain reaction [PCR method]) were also performed at RIKEN Cell Bank (Tsukuba, Japan). Identification of mouse strain (Simple sequence length polymorphism analysis), identification of animal species (PCR method), morphology, cell viability, and adhesion efficiency had performed for authentication according to ICLAC. Cells were maintained in RPMI-1640 (Sigma) supplemented with 5 % (vol/vol) fetal bovine serum (FBS) (Thermo Fisher). To inoculate the tumor cells into mice, cells were harvested by trypsinization, washed with sterile PBS, and injected intradermally (2 × 10^5^ cells/50 μl sterile PBS/mouse) on the flank of each mouse. Tumor growth was measured every three or 4 days by using a caliper.

### Cell preparations

For tumor-infiltrating Treg cell preparation, tumor tissues were harvested 3 weeks after tumor inoculation. Tumor tissues were cut into small pieces, incubated in 5% FBS RPMI-1640 in the presence of an enzyme mixture (Miltenyi Biotec) at 37℃ for 45 min, and digested by using a gentleMACS Dissociator and tumor dissociation kit (Miltenyi Biotec), according to the manufacturer’s instructions. Cells were filtered through 70 μm nylon mesh and subsequently centrifuged using different concentrations of Percoll (Sigma-Aldrich) to exclude tissue debris and were washed with staining medium.

BMDCs were generated as described previously (Nakahashi-Oda et al., 2016). Briefly, bone marrow cells were cultured in a 10 cm culture dish in complete RPMI-1640 containing 10 % FBS in the presence of 10 ng/ml GM-CSF (WAKO) and 10 ng/ml IL-4 (WAKO) for 7 days. BMDCs were enriched by using CD11c MACS Beads (Miltenyi Biotec) to remove dead cells generated during BMDC development.

### Cytokine production from tumor-infiltrating lymphocytes

Cells were isolated from tumors in mice 3 weeks after inoculation, and stimulated for 4 hr with 50 ng/ml PMA and 500 ng/ml ionomycin. Brefeldin A (Sigma-Aldrich) was added for the last 3 hr of culture. Cells were treated by using Foxp3 staining kits (eBioscience) and then stained with anti-IFN-γmAb.

### Immunohistochemistry and immunocytochemical staining

Paraffin-embedded tumor samples were deparaffinized in xylene and a series of graded concentrations of alcohol. To block endogenous horseradish peroxidase (HRP), tissue sections were incubated in 0.3 % hydrogen peroxidase in methanol for 30 min at room temperature. For antigen retrieval, the specimens were preheated in AR6 buffer (PerkinElmer). Samples were incubated with anti-GFP (D5.1) XP (Cell signaling) or Rat anti-Foxp3 (FJK-16s; Thermo Fisher) for 1 hr at room temperature or overnight at 4 °C, respectively, and then incubated with appropriate secondary HRP-conjugated Abs. An HRP-conjugated dextran polymer system (PerkinElmer) was used for detection. After being washed with TBST, sections were mounted with 4′,6-diamidino-2-phenylindole (DAPI; Vector labs). For quantification of Foxp3+ cells in tumor tissues, tissue sections were scanned using BZ-X710 (Keyence). The number of Foxp3+ cells per high-power field in each area was automatically counted with hybrid cell counts software (Keyence). For immunocytochemical staining, 1.0 × 10^5^ BMDCs were cultured in eight-well chamber slides (Thermo Fisher) and were stimulated with pHrodo Red ester or pHrodo STP Green (Thermo Fisher)-labeled exosomes. Cells were then fixed with 10 % paraformaldehyde at 4°C for 20 min, permeabilized with 0.3 % Triton-X, and then stained with a mAb to EEA-1 (1G11; eBioscience) or TLR3 (11F8; Biolegend), followed by Alexa Flor 488-conjugated donkey anti-mouse IgG or Alexa Flor 546-conjugated goat anti-rat IgG (Invitrogen), respectively. Samples were evaluated by use of laser scanning confocal microscopy (FV10i FLOUVIEW; Olympus).

### In vivo depletion of Treg cells

For in vivo depletion of Treg cells, mice were injected intraperitoneally with 300 μg of an anti-CD25 mAb (PC61) and an isotype control Ab on days −6, −3, and 0 before B16 tumor inoculation.

### EV inhibitor treatment

To inhibit EV generation, mice were injected with 1.0 mg/kg GW4869 (Ikebuchi et al., 2018; Kosaka et al., 2013) (Cayman Chemical) intratumorally on days 14, 18, and 21 after tumor inoculation. Tumor tissues were harvested on day 25.

### EV isolation and treatment

B16 melanoma cells were cultured in complete RPMI supplemented with or without 2 % bovine serum albumin. The culture medium was harvested and subjected to sequential centrifugation steps (first, 5 min for 2000 G; second, 20 min for 10,000 G). EVs were purified by using an Exosome Isolation Kit (WAKO) according to the manufacturer’s protocol. In brief, streptavidin magnetic beads, bound with biotinylated mouse Tim4-Fc, which is the phosphatidylserine receptors, were added to the culture medium of B16 melanoma containing 2 mM CaCl_2_, and the mixture was rotated for 3 hr or overnight at 4 °C. The beads were washed three times with washing buffer and exosomes were eluted with elution buffer (Figure 3D). For quantification of the EVs in the elution buffer, the concentration of EV protein was quantified by using a BCA Protein Assay Kit (Novagen). For BMDC stimulation by EVs, 2 × 10^5^ BMDCs were incubated in the presence of 3–5 μg/ml EVs for 2.5 h. To inhibit TLR3 and TLR4 signaling, a TLR3/dsRNA complex inhibitor (Merck) and a TLR4 inhibitor (TAK-242; Merck) were added to the cultures of BMDCs for 15 min before exosome stimulation.

### Coculture of iTreg cells with EV-stimulated BMDCs

CD4+ T cells were enriched from the spleen cells by using mouse CD4 MACS Beads (L3T4, Miltenyi Biotec) and then CD4^+^CD44^lo^CD62L^high^Foxp3-eGFP^−^ naive T cells were purified by sorting with flow cytometry (FACS Aria III, Becton Dickinson). Inducible Treg cells were generated by culture of naive CD4^+^ T cells in the presence of plate-coated 0.33 μg/ml anti-CD3 Ab (145–2 C11; TONBO), 2.0 μg/ml soluble CD28 (37.51; Biolegend), 20 ng/ml IL-2 (BD Pharmingen), and 2.5 ng/ml TGF-β (R&D system) for 3 days. Inducible Treg cells (5 × 10^4^ cells/well) were cultured with exosome-stimulated BMDCs (5 × 10^4^ cells/well) in 96-well round-bottom plates in the presence of IL-2 and TGF-β for 5 days.

### Quantitative real-time PCR analysis

Total RNA was extracted from tumor-infiltrating CD11c + cells and BMDCs. Reverse transcription was performed with a High-Capacity cDNA Reverse Transcription Kit (Applied Biosystems). Quantitative PCR analysis was performed with Power SYBER Green PCR Master Mix (Applied Biosystem) by using an ABI 7500 sequence detector (Applied Biosystems). The PCR primers are as follows: *Ifnb* fwd, 5′-cagctccaagaaaggacgaac-3′; *Ifnb* rev, 5′-ggcagtgtaactcttctgcat-3′; *Il10* fwd, 5′-gctggacaacatactgctaacc-3′; *Il10* rev, 5′- atttccgataaggcttggcaa-3′; and *Tgfb* fwd, 5′-tgacgtcactggagttgtacgg-3′; *Tgfb* rev, 5′-ggttcatgtcatggatggtgc-3′; normalization of quantitative real-time PCR was performed based on the gene encoding β-actin.

### Western blots

BMDCs were stimulated or unstimulated with exosomes for 20 or 40 min and lysed with 1% NP-40. The lysetes of BMDCs were immunoblotted with antibody to phosphorylated IRF3 (4D4G; Cell Signaling Technology) or IRF3 (FL-425; Santa Cruz Biotechnology).

### Bioinformatics

For analysis of melanoma scRNA-seq, data were downloaded from the database of scRNA-seq analysis of melanoma (accession no. GSE72056). The matrix data were passed to the R software package Seurat. Cells that had unique gene counts of <200 were excluded, as were all genes that were expressed in >3 cells. Counted data were log2-transformed and scaled by Seurat’s *Scale Data* function. Principal component (PC) analysis was performed on a set of highly variable genes defined by Seurat’s *FindVariableGenes* function. Genes associated the resulting PCs were then used for dimensionality reduction by using *t*-distributed stochastic neighbor embedding. Cluster-based marker identification and differential expression were performed using Seurat’s *FindAllmarkers*. RNA-seq and survival data were obtained from TCGA project and analyzed by using OncoLnc and GEPIA (Anaya, 2016; Tang et al., 2017).

### Statistical analyses

Comparisons were performed using GraphPad Prism version 5.0 (GraphPad Software) by one- or two-way analysis of variance, followed by Bonferroni’s multiple comparisons test or Student’s unpaired *t*-test. Data are presented as means ± standard error of the means, and differences are considered significant at p < 0.05.

## Data Availability

All data generated or analysed during this study are included in the manuscript and supporting files. Source data files have been provided for Figures 1 to 5 and figure supplements for Figures 2 to 5. The following previously published datasets were used: TiroshI IzarB PrakadanS WadsworthM TreacyD TrombettaJ RotemA RodmanC LianC MurphyG Fallahi-SichaniM Dutton-RegesterK LinJ CohenO ShahP LuD GenshaftA ShalekAK RegevA GarrawayLA 2016Single cell RNA-seq analysis of melanomaNCBI Gene Expression OmnibusGSE72056

## Additional files

Transparent reporting form

## References

[bib1] Adeegbe DO, Nishikawa H (2013). Natural and Induced T Regulatory Cells in Cancer. Frontiers in Immunology.

[bib2] Anaya J (2016). OncoLnc: linking TCGA survival data to mRNAs, miRNAs, and lncRNAs. PeerJ Computer Science.

[bib3] Andreola G, Rivoltini L, Castelli C, Huber V, Perego P, Deho P, Squarcina P, Accornero P, Lozupone F, Lugini L, Stringaro A, Molinari A, Arancia G, Gentile M, Parmiani G, Fais S (2002). Induction of lymphocyte apoptosis by tumor cell secretion of FasL-bearing microvesicles. The Journal of Experimental Medicine.

[bib4] Bedoui S, Whitney PG, Waithman J, Eidsmo L, Wakim L, Caminschi I, Allan RS, Wojtasiak M, Shortman K, Carbone FR, Brooks AG, Heath WR (2009). Cross-presentation of viral and self antigens by skin-derived CD103+ dendritic cells. Nature Immunology.

[bib5] Borrego F (2013). The CD300 molecules: an emerging family of regulators of the immune system. Blood.

[bib6] Chen G, Huang AC, Zhang W, Zhang G, Wu M, Xu W, Yu Z, Yang J, Wang B, Sun H, Xia H, Man Q, Zhong W, Antelo LF, Wu B, Xiong X, Liu X, Guan L, Li T, Liu S, Yang R, Lu Y, Dong L, McGettigan S, Somasundaram R, Radhakrishnan R, Mills G, Lu Y, Kim J, Chen YH, Dong H, Zhao Y, Karakousis GC, Mitchell TC, Schuchter LM, Herlyn M, Wherry EJ, Xu X, Guo W (2018). Exosomal PD-L1 contributes to immunosuppression and is associated with anti-PD-1 response. Nature.

[bib7] Cheng K, Wang X, Yin H (2011). Small-molecule inhibitors of the TLR3/dsRNA complex. Journal of the American Chemical Society.

[bib8] Clark GJ, Cooper B, Fitzpatrick S, Green BJ, Hart DN (2001). The gene encoding the immunoregulatory signaling molecule CMRF-35A localized to human chromosome 17 in close proximity to other members of the CMRF-35 family. Tissue Antigens.

[bib9] Clayton A, Mitchell JP, Court J, Mason MD, Tabi Z (2007). Human tumor-derived exosomes selectively impair lymphocyte responses to interleukin-2. Cancer Research.

[bib10] Couto N, Caja S, Maia J, Strano Moraes MC, Costa-Silva B (2018). Exosomes as emerging players in cancer biology. Biochimie.

[bib11] Fleming V, Hu X, Weller C, Weber R, Groth C, Riester Z, Hüser L, Sun Q, Nagibin V, Kirschning C, Bronte V, Utikal J, Altevogt P, Umansky V (2019). Melanoma Extracellular Vesicles Generate Immunosuppressive Myeloid Cells by Upregulating PD-L1 via TLR4 Signaling. Cancer Research.

[bib12] Francisco LM, Salinas VH, Brown KE, Vanguri VK, Freeman GJ, Kuchroo VK, Sharpe AH (2009). PD-L1 regulates the development, maintenance, and function of induced regulatory T cells. The Journal of Experimental Medicine.

[bib13] Gangaplara A, Martens C, Dahlstrom E, Metidji A, Gokhale AS, Glass DD, Lopez-Ocasio M, Baur R, Kanakabandi K, Porcella SF, Shevach EM (2018). Type I interferon signaling attenuates regulatory T cell function in viral infection and in the tumor microenvironment. PLOS Pathogens.

[bib14] Gao Y, Nish SA, Jiang R, Hou L, Licona-Limón P, Weinstein JS, Zhao H, Medzhitov R (2013). Control of T Helper 2 Responses by Transcription Factor IRF4-Dependent Dendritic Cells. Immunity.

[bib15] Grange C, Tapparo M, Collino F, Vitillo L, Damasco C, Deregibus MC, Tetta C, Bussolati B, Camussi G (2011). Microvesicles released from human renal cancer stem cells stimulate angiogenesis and formation of lung premetastatic niche. Cancer Research.

[bib16] Hsu P, Santner-Nanan B, Hu M, Skarratt K, Lee CH, Stormon M, Wong M, Fuller SJ, Nanan R (2015). IL-10 Potentiates Differentiation of Human Induced Regulatory T Cells via STAT3 and Foxo1. Journal of Immunology.

[bib17] Huber V, Vallacchi V, Fleming V, Hu X, Cova A, Dugo M, Shahaj E, Sulsenti R, Vergani E, Filipazzi P, De Laurentiis A, Lalli L, Di Guardo L, Patuzzo R, Vergani B, Casiraghi E, Cossa M, Gualeni A, Bollati V, Arienti F, De Braud F, Mariani L, Villa A, Altevogt P, Umansky V, Rodolfo M, Rivoltini L (2018). Tumor-derived microRNAs induce myeloid suppressor cells and predict immunotherapy resistance in melanoma. The Journal of Clinical Investigation.

[bib18] Ikebuchi Y, Aoki S, Honma M, Hayashi M, Sugamori Y, Khan M, Kariya Y, Kato G, Tabata Y, Penninger JM, Udagawa N, Aoki K, Suzuki H (2018). Coupling of bone resorption and formation by RANKL reverse signalling. Nature.

[bib19] Kim JM, Rasmussen JP, Rudensky AY (2007). Regulatory T cells prevent catastrophic autoimmunity throughout the lifespan of mice. Nature Immunology.

[bib20] Klages K, Mayer CT, Lahl K, Loddenkemper C, Teng MWL, Ngiow SF, Smyth MJ, Hamann A, Huehn J, Sparwasser T (2010). Selective Depletion of Foxp3+ Regulatory T Cells Improves Effective Therapeutic Vaccination against Established Melanoma. Cancer Research.

[bib21] Kosaka N, Iguchi H, Yoshioka Y, Takeshita F, Matsuki Y, Ochiya T (2010). Secretory Mechanisms and Intercellular Transfer of MicroRNAs in Living Cells. The Journal of Biological Chemistry.

[bib22] Kosaka N, Iguchi H, Hagiwara K, Yoshioka Y, Takeshita F, Ochiya T (2013). Neutral sphingomyelinase 2 (nSMase2)-dependent exosomal transfer of angiogenic microRNAs regulate cancer cell metastasis. The Journal of Biological Chemistry.

[bib23] Lima LG, Chammas R, Monteiro RQ, Moreira MEC, Barcinski MA (2009). Tumor-derived microvesicles modulate the establishment of metastatic melanoma in a phosphatidylserine-dependent manner. Cancer Letters.

[bib24] Liu Y, Gu Y, Han Y, Zhang Q, Jiang Z, Zhang X, Huang B, Xu X, Zheng J, Cao X (2016). Tumor Exosomal RNAs Promote Lung Pre-metastatic Niche Formation by Activating Alveolar Epithelial TLR3 to Recruit Neutrophils. Cancer Cell.

[bib25] Medrano RFV, Hunger A, Mendonça SA, Barbuto JAM, Strauss BE (2017). Immunomodulatory and antitumor effects of type I interferons and their application in cancer therapy. Oncotarget.

[bib26] Melo SA, Luecke LB, Kahlert C, Fernandez AF, Gammon ST, Kaye J, LeBleu VS, Mittendorf EA, Weitz J, Rahbari N, Reissfelder C, Pilarsky C, Fraga MF, Piwnica-Worms D, Kalluri R (2015). Glypican-1 identifies cancer exosomes and detects early pancreatic cancer. Nature.

[bib27] Metidji A, Rieder SA, Glass DD, Cremer I, Punkosdy GA, Shevach EM (2015). IFN-α/β receptor signaling promotes regulatory T cell development and function under stress conditions. Journal of Immunology.

[bib28] Minn AJ (2015). Interferons and the Immunogenic Effects of Cancer Therapy. Trends in Immunology.

[bib29] Morimoto Y, Kishida T, Kotani S-I, Takayama K, Mazda O (2018). Interferon-β signal may up-regulate PD-L1 expression through IRF9-dependent and independent pathways in lung cancer cells. Biochemical and Biophysical Research Communications.

[bib30] Mougiakakos D, Johansson CC, Trocme E, All-Ericsson C, Economou MA, Larsson O, Seregard S, Kiessling R (2010). Intratumoral forkhead box P3-positive regulatory T cells predict poor survival in cyclooxygenase-2-positive uveal melanoma. Cancer.

[bib31] Muller L, Simms P, Hong CS, Nishimura MI, Jackson EK, Watkins SC, Whiteside TL (2017). Human tumor-derived exosomes (TEX) regulate Treg functions via cell surface signaling rather than uptake mechanisms. Oncoimmunology.

[bib32] Nakahashi-Oda C, Tahara-Hanaoka S, Honda S, Shibuya K, Shibuya A (2012a). Identification of phosphatidylserine as a ligand for the CD300a immunoreceptor. Biochemical and Biophysical Research Communications.

[bib33] Nakahashi-Oda C, Tahara-Hanaoka S, Shoji M, Okoshi Y, Nakano-Yokomizo T, Ohkohchi N, Yasui T, Kikutani H, Honda S, Shibuya K, Nagata S, Shibuya A (2012b). Apoptotic cells suppress mast cell inflammatory responses via the CD300a immunoreceptor. The Journal of Experimental Medicine.

[bib34] Nakahashi-Oda C, Udayanga KGS, Nakamura Y, Nakazawa Y, Totsuka N, Miki H, Iino S, Tahara-Hanaoka S, Honda S, Shibuya K, Shibuya A (2016). Apoptotic epithelial cells control the abundance of Treg cells at barrier surfaces. Nature Immunology.

[bib35] Nishikawa H, Sakaguchi S (2010). Regulatory T cells in tumor immunity. Ternational Journal of Cancer.

[bib36] Ondondo B, Jones E, Godkin A, Gallimore A (2013). Home sweet home: the tumor microenvironment as a haven for regulatory T cells. Frontiers in Immunology.

[bib37] Onizuka S, Tawara I, Shimizu J, Sakaguchi S, Fujita T, Nakayama E (1999). Tumor rejection by in vivo administration of anti-CD25 (interleukin-2 receptor alpha) monoclonal antibody. Cancer Research.

[bib38] Patidar A, Selvaraj S, Sarode A, Chauhan P, Chattopadhyay D, Saha B (2018). DAMP-TLR-cytokine axis dictates the fate of tumor. Cytokine.

[bib39] Ramsdell F, Ziegler SF (2014). FOXP3 and scurfy: how it all began. Nature Reviews. Immunology.

[bib40] Sarkar SN, Peters KL, Elco CP, Sakamoto S, Pal S, Sen GC (2004). Novel roles of TLR3 tyrosine phosphorylation and PI3 kinase in double-stranded RNA signaling. Nature Structural & Molecular Biology.

[bib41] Sawant DV, Yano H, Chikina M, Zhang Q, Liao M, Liu C, Callahan DJ, Sun Z, Sun T, Tabib T, Pennathur A, Corry DB, Luketich JD, Lafyatis R, Chen W, Poholek AC, Bruno TC, Workman CJ, Vignali DAA (2019). Adaptive plasticity of IL-10+ and IL-35+ Treg cells cooperatively promotes tumor T cell exhaustion. Nature Immunology.

[bib42] Shitara K, Nishikawa H (2018). Regulatory T cells: a potential target in cancer immunotherapy. Annals of the New York Academy of Sciences.

[bib43] Skog J, Würdinger T, van Rijn S, Meijer DH, Gainche L, Sena-Esteves M, Curry WT, Carter BS, Krichevsky AM, Breakefield XO (2008). Glioblastoma microvesicles transport RNA and proteins that promote tumour growth and provide diagnostic biomarkers. Nature Cell Biology.

[bib44] Snell LM, McGaha TL, Brooks DG (2017). Type I Interferon in Chronic Virus Infection and Cancer. Trends in Immunology.

[bib45] Srivastava S, Koch MA, Pepper M, Campbell DJ (2014). Type I interferons directly inhibit regulatory T cells to allow optimal antiviral T cell responses during acute LCMV infection. The Journal of Experimental Medicine.

[bib46] Szajnik M, Czystowska M, Szczepanski MJ, Mandapathil M, Whiteside TL (2010). Tumor-derived microvesicles induce, expand and up-regulate biological activities of human regulatory T cells (Treg). PLOS ONE.

[bib47] Tang Z, Li C, Kang B, Gao G, Li C, Zhang Z (2017). GEPIA: a web server for cancer and normal gene expression profiling and interactive analyses. Nucleic Acids Research.

[bib48] Tatematsu M, Nishikawa F, Seya T, Matsumoto M (2013). Toll-like receptor 3 recognizes incomplete stem structures in single-stranded viral RNA. Nature Communications.

[bib49] Tian X, Shen H, Li Z, Wang T, Wang S (2019). Tumor-derived exosomes, myeloid-derived suppressor cells, and tumor microenvironment. Journal of Hematology & Oncology.

[bib50] van Niel G, D’Angelo G, Raposo G (2018). Shedding light on the cell biology of extracellular vesicles. Nature Reviews Molecular Cell Biology.

[bib51] Voss OH, Tian L, Murakami Y, Coligan JE, Krzewski K (2015). Emerging role of CD300 receptors in regulating myeloid cell efferocytosis. Molecular & Cellular Oncology.

[bib52] Wan YY, Flavell RA (2007). “Yin-Yang” functions of transforming growth factor-beta and T regulatory cells in immune regulation. Immunological Reviews.

[bib53] Wang X, Luo G, Zhang K, Cao J, Huang C, Jiang T, Liu B, Su L, Qiu Z (2018). Hypoxic Tumor-Derived Exosomal miR-301a Mediates M2 Macrophage Polarization via PTEN/PI3Kγ to Promote Pancreatic Cancer Metastasis. Cancer Research.

[bib54] Wang Y, Nakahashi-Oda C, Okayama Y, Shibuya A (2019). Autonomous regulation of IgE-mediated mast cell degranulation and immediate hypersensitivity reaction by an inhibitory receptor CD300a. The Journal of Allergy and Clinical Immunology.

[bib55] Wieckowski EU, Visus C, Szajnik M, Szczepanski MJ, Storkus WJ, Whiteside TL (2009). Tumor-Derived Microvesicles Promote Regulatory T Cell Expansion and Induce Apoptosis in Tumor-Reactive Activated CD8+ T Lymphocytes. Journal of Immunology.

[bib56] Witwer KW, Théry C (2019). Extracellular vesicles or exosomes? On primacy, precision, and popularity influencing a choice of nomenclature. Journal of Extracellular Vesicles.

[bib57] Xiao W, Klement JD, Lu C, Ibrahim ML, Liu K (2018). IFNAR1 Controls Autocrine Type I IFN Regulation of PD-L1 Expression in Myeloid-Derived Suppressor Cells. Journal of Immunology.

[bib58] Ying X, Wu Q, Wu X, Zhu Q, Wang X, Jiang L, Chen X, Wang X (2016). Epithelial ovarian cancer-secreted exosomal miR-222-3p induces polarization of tumor-associated macrophages. Oncotarget.

[bib59] Yotsumoto K, Okoshi Y, Shibuya K, Yamazaki S, Tahara-Hanaoka S, Honda SI, Osawa M, Kuroiwa A, Matsuda Y, Tenen DG, Iwama A, Nakauchi H, Shibuya A (2003). Paired activating and inhibitory immunoglobulin-like receptors, MAIR-I and MAIR-II, regulate mast cell and macrophage activation. The Journal of Experimental Medicine.

[bib60] Zebrowska A, Widlak P, Whiteside T, Pietrowska M (2020). Signaling of Tumor-Derived sEV Impacts Melanoma Progression. Ternational Journal of Molecular Sciences.

[bib61] Zitvogel L, Galluzzi L, Kepp O, Smyth MJ, Kroemer G (2015). Type I interferons in anticancer immunity. Nature Reviews. Immunology.

